# Mitochondrial oxidative stress activates COX-2/mPGES-1/PGE2 cascade induced by albumin in renal proximal tubular cells

**DOI:** 10.18632/oncotarget.24187

**Published:** 2018-01-12

**Authors:** Yibo Zhuang, Chenhu Wang, Chunfeng Wu, Dan Ding, Fei Zhao, Caiyu Hu, Wei Gong, Guixia Ding, Yue Zhang, Lihong Chen, Guangrui Yang, Chunhua Zhu, Aihua Zhang, Zhanjun Jia, Songming Huang

**Affiliations:** ^1^ Department of Nephrology, Children’s Hospital of Nanjing Medical University, Nanjing 210008, China; ^2^ Jiangsu Key Laboratory of Pediatrics, Nanjing 210029, China; ^3^ Nanjing Key Laboratory of Pediatrics, Nanjing 210008, China

**Keywords:** albumin, mitochondrial oxidative stress, COX-2, PGE2, proximal tubular cells

## Abstract

COX-2/mPGES-1/PGE2 cascade is of importance in the pathogenesis of kidney injury. Meanwhile, recent studies documented a detrimental role of mitochondrial oxidative stress in kidney diseases. The present study was undertaken to investigate the role of mitochondrial oxidative stress in albumin-induced activation of COX-2/mPGES-1/PGE2 cascade in renal proximal tubular cells. Following albumin overload in mice, we observed a significant increase of oxidative stress and mitochondrial abnormality determined by transmission electron microscope, which was attenuated by the administration of MnTBAP, a mitochondrial SOD2 mimic. More interestingly, albumin overload-induced upregulation of COX-2 and mPGES-1 at mRNA and protein levels was largely abolished by MnTBAP treatment in mice. Meanwhile, urinary PGE2 excretion was also blocked by MnTBAP treatment. Furthermore, mouse proximal tubule epithelial cells (mPTCs) were treated with albumin. Similarly, COX-2/mPGES-1/PGE2 cascade was significantly activated by albumin in dose- and time-dependent manners, which was abolished by MnTBAP treatment in parallel with a blockade of oxidative stress. Collectively, the findings from current study demonstrated that mitochondrial oxidative stress could activate COX-2/mPGES-1/PGE2 cascade in proximal tubular cells under the proteinuria condition. Mitochondrial oxidative stress/COX-2/mPGES-1/PGE2 could serve as the important targets for the treatment of proteinuria-associated kidney injury.

## INTRODUCTION

Proteinuria plays an established role in mediating renal tubular injury and is viewed as a causative factor in promoting the progression of kidney diseases [1, 2]. Evidence from numerous studies suggested that the cellular phenotypic changes and apoptotic response may serve as the underlying mechanisms of proteinuria-induced kidney injury [3, 4]. However, detailed molecular mechanisms remain elusive.

PGE2 is generated through a cyclooxygenases (COXs)/PGE2 synthases (PGESs) cascade [5]. To date, three PGE2 synthases including mPGES-1, mPGES-2, and cPGES were cloned with the best characterization of mPGES-1. The genetic deletion of mPGES-2 and cPGES did not reduce PGE2 production under basal or stress conditions [6, 7]. Kidney is a major source of prostaglandins (PGs) including PGE2, PGD2, PGI2, PGF2α, and thromboxane A2 (TXA2). PGE2 is of importance in fluid metabolism regulation [8–11] and kidney injuries [12–14]. PGI2 and TXA2 are documented to be important in modulating renal hemodynamics [15, 16]. The roles of PGD2 and PGF2α in kidney remain uncertain. Our previous reports demonstrated that COX-2 and mPGES-1 played detrimental role in CKD model of 5/6 nephrectomy [17] and AKI model of cisplatin nephropathy [18]. In diabetic kidney disease, blockade of COX-2 or PGE2 receptors of EP1 and EP4 resulted in significant protection against diabetic kidney injury [19]. In another proteinuric animal model induced by adriamycin, the overexpression of COX-2 in podocytes remarkably worsened kidney damage [20]. All of these data suggest a critical role of the COX-2/mPGES-1/PGE2 cascade in mediating kidney injury.

Mitochondrial abnormality has been found in albumin-treated human proximal tubule cells [21] and CKDs [22, 23]. Moreover, our previous studies also demonstrated that mitochondrial oxidative stress contributes to the kidney injury induced by the aldosterone [24, 25]. These findings highly suggested a detrimental role of mitochondrial dysfunction in proteinuric kidney diseases. Thus, in the present study, we investigated the role of mitochondrial oxidative stress in albuminuria-stimulated activation of COX-2/mPGES-1/PGE2 cascade *in vivo* and *in vitro*.

## RESULTS

### Albumin overload-induced mitochondrial abnormality and oxidative stress was ameliorated by MnTBAP

**Figure 1:**
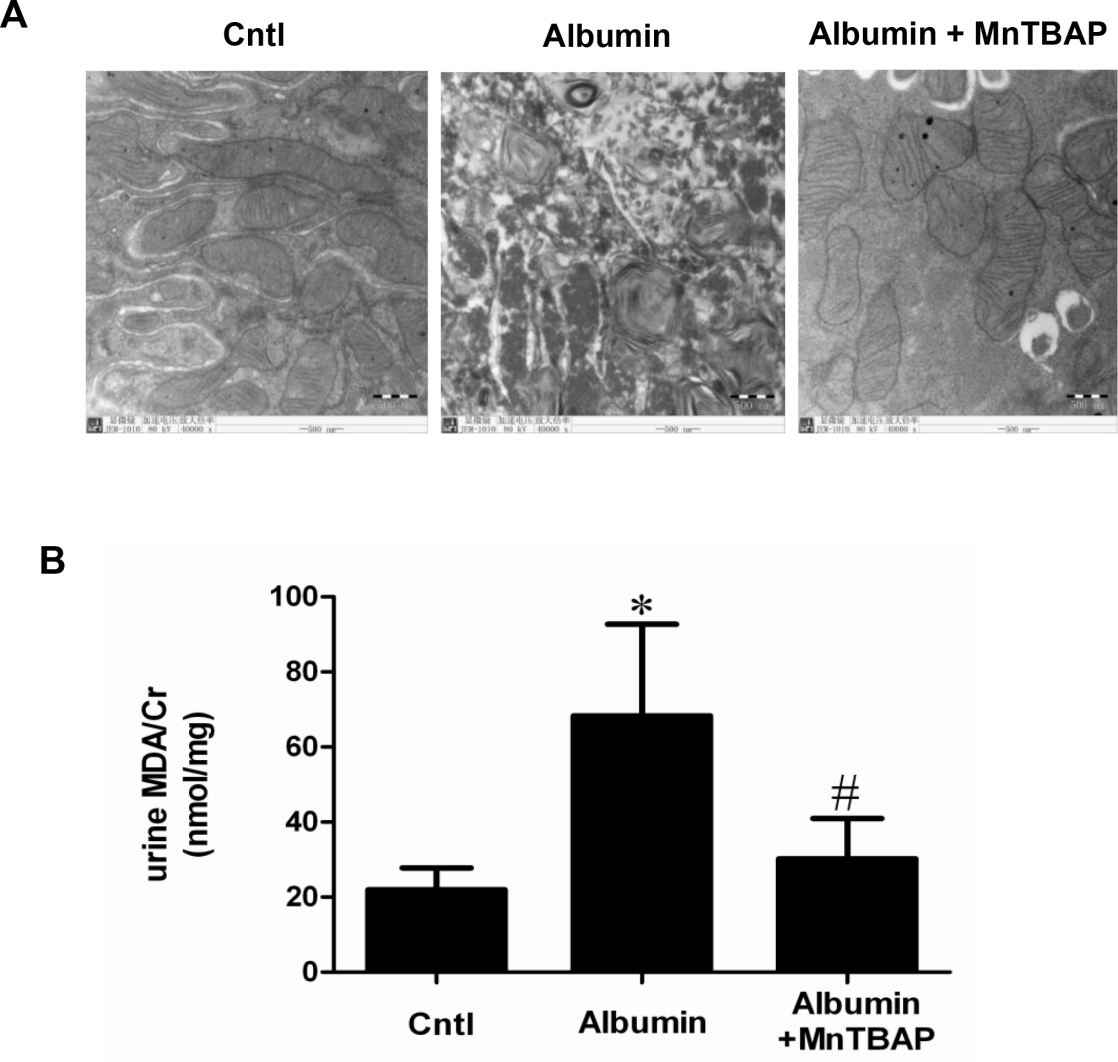
MnTBAP treatment ameliorated oxidative stress and mitochondrial abnormality induced by albumin overload in mice (**A**) Mitochondrial morphology in tubular epithelial cells determined by TEM. (**B**) Renal TBARs levels. The values represent means ± SD (*n* = 8). ^*^P < 0.01 vs. control. ^#^*P* < 0.01 vs. albumin-overload mice.

Eleven days of albumin overload in mice resulted in a severe structural disruption of the mitochondria in renal tubular cells as determined by TEM (Figure 1A). Meanwhile, the oxidative stress marker of MDA in urine was elevated by albumin overload (Figure 1B). Strikingly, all these abnormalities were reversed by a mitochondrial SOD2 mimic MnTBAP (Figure 1A and 1B).

### Activation of COX-2/mPGES-1/PGE2 cascade by albumin overload was blocked by MnTBAP in mice

**Figure 2:**
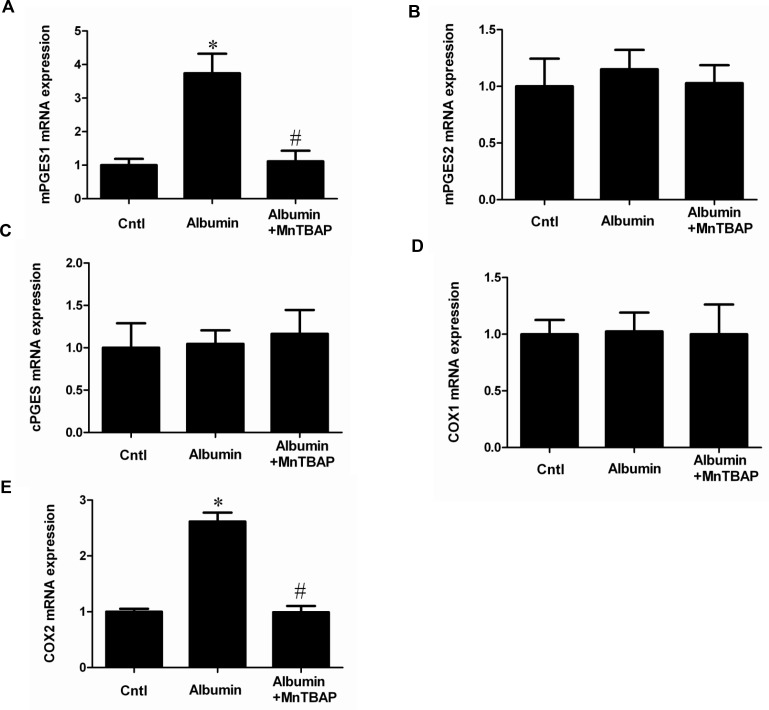
The upregulation of COX-2 and mPGES-1 was revered by MnTBAP at mRNA levels (**A**) qRT-PCR analysis of mPGES-1. (**B**) qRT-PCR analysis of mPGES-2. (**C**) qRT-PCR analysis of cPGES. (**D**) qRT-PCR analysis of COX-1. (**E**) qRT-PCR analysis of COX-2. The values represent means ± SD (*n* = 8). ^*^P < 0.01 vs. control. ^#^*P <* 0.01 vs. albumin-overload mice.

**Figure 3:**
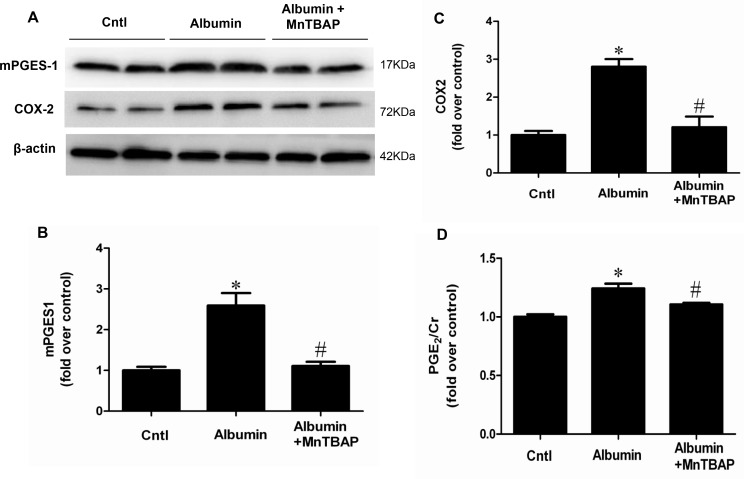
MnTBAP treatment reversed the effects of albumin overload on the protein expressions of COX-2 and mPGES-1 and urinary PGE2 production (**A**) Western blots of mPGES-1, COX-2 and β-actin. (**B**) Densitometric analysis of mPGES-1. (**C**) Densitometric analysis of COX-2. (**D**) Urinary PGE2 output. The values represent means ± SD (*n* = 8). ^*^p < 0.01 vs. control. ^#^*p* < 0.01 vs. albumin-overload mice.

By qRT-PCR, we observed a selective upregulation of COX-2 and mPGES-1 but not COX-1, mPGES-2, and cPGES (Figure 2A–2E). By Western blotting, we further confirmed the up-regulation of COX-2 and mPGES-1 at the protein levels (Figure 3A–3C). Moreover, albumin overload significantly increased urinary PGE2 excretion (Figure 3D). Interestingly, application of MnTBAP largely normalized the stimulation of COX-2/mPGE-1/PGE2 cascade (Figure 3A–3D) without the impact on other PGE2 synthetic enzymes (Figure 2B–2D). These data suggested that albumin overload-induced oxidative stress might serve as a key factor leading to the stimulation of COX-2/mPGES-1/PGE2 cascade.

### Albumin directly stimulated COX-2/mPGES-1/PGE2 cascade in renal proximal tubular cells

**Figure 4:**
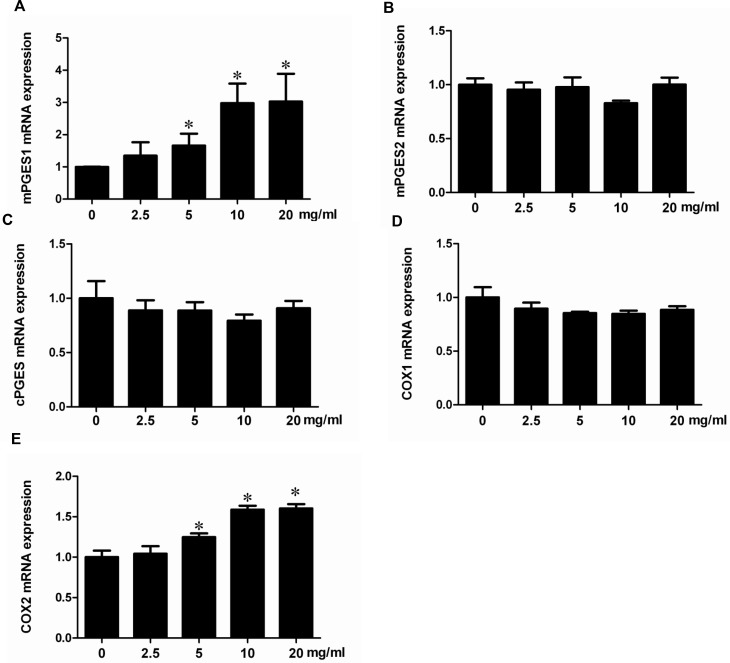
Albumin upregulated the mRNA expressions of COX-2 and mPGES-1 in a dose-dependent manner in mPTCs (**A**) qRT-PCR analysis of mPGES-1. (**B**) qRT-PCR analysis of mPGES-2. (**C**) qRT- PCR analysis of cPGES. (**D**) qRT-PCR analysis of COX-1. (**E**) qRT-PCR analysis of COX-2. The values represent means ± SD (*n* = 6). ^*^P < 0.01 vs. control.

**Figure 5:**
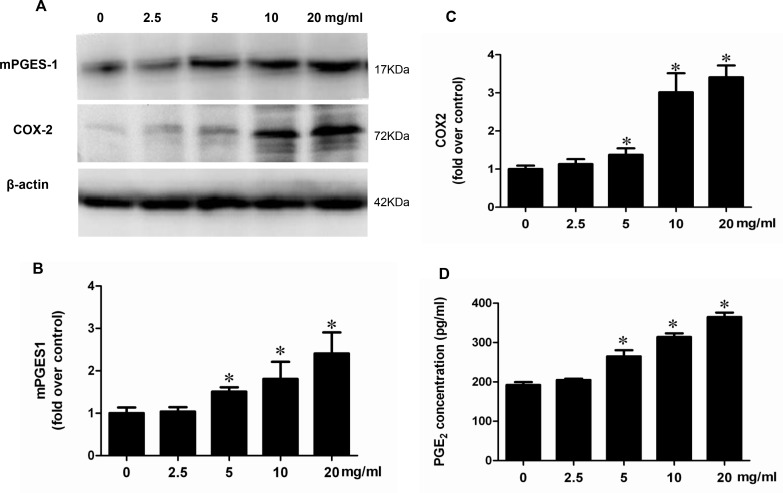
Albumin stimulated the protein expressions of COX-2 and mPGES-1 and PGE2 release in a dose-dependent manner in mPTCs (**A**) Western blots of mPGES-1, COX-2, and β-actin. (**B**) Densitometric analysis of mPGES-1. (**C**) Densitometric analysis of COX-2. (**D**) PGE2 concentration in medium. The values represent means ± SD (*n* = 6).

**Figure 6:**
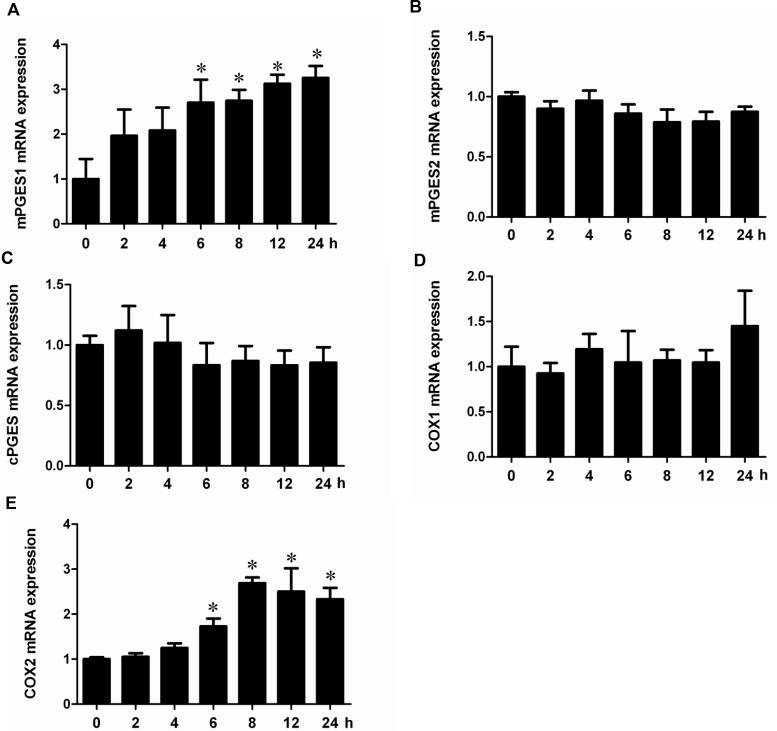
Albumin upregulated the mRNA expressions of COX-2 and mPGES-1 in a time-dependent manner in mPTCs (**A**) qRT-PCR analysis of mPGES-1. (**B**) qRT-PCR analysis of mPGES-2. (**C**) qRT- PCR analysis of cPGES. (**D**) qRT-PCR analysis of COX-1. (**E**) qRT-PCR analysis of COX-2. The values represent means ± SD (*n* = 6). ^*^P < 0.01 vs. control.

**Figure 7:**
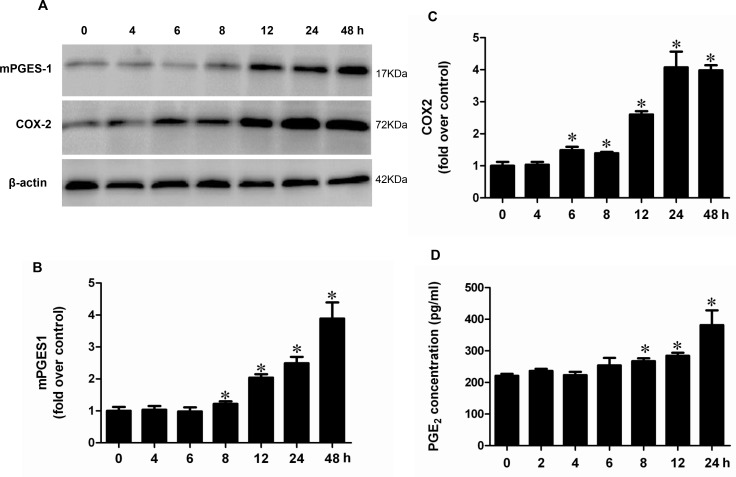
Albumin stimulated the protein expressions of COX-2 and mPGES-1 and PGE2 release in a time-dependent manner in mPTCs (**A**) Western blots of mPGES-1, COX-2, and β-actin. (**B**) Densitometric analysis of mPGES-1. (**C**) Densitometric analysis of COX-2. (**D**) PGE2 concentration in medium. The values represent means ± SD (*n* = 6). ^*^P < 0.01 vs. control.

To investigate whether albumin could directly stimulate COX-2/mPGES-1/PGE2 cascade *in vitro*, the mPTCs were treated with albumin, and the regulation of PGE2 synthetic enzymes including COX-2, COX-1, mPGES-1, mPGES-2, and cPGES was determined by qRT-PCR and Western blotting. As shown by the data, albumin dose-dependently induced COX-2, mPGES-1 mRNA (Figure 4A–4E) and protein (Figure 5A–5C) expressions without affecting the other PGE2 synthetic enzymes. Meanwhile, a time-dependent stimulation of this COX-2/mPGES-1 pathway was also observed at mRNA (Figure 6A–6E) and protein (Figure 7A–7C) levels. In line with the stimulation of the COX-2/mPGES-1 axis, the PGE2 release in the cell culture medium was also dose- and time-dependently elevated (Figure 5D and Figure7D). All of these data indicated a direct effect of albumin on the stimulation of COX-2/mPGES-1/PGE2 cascade in renal proximal tubular cells.

### Inhibition of mitochondrial oxidative stress abolished albumin-induced activation of the COX-2/mPGES-1/PGE2 cascade in renal proximal tubular cells

**Figure 8:**
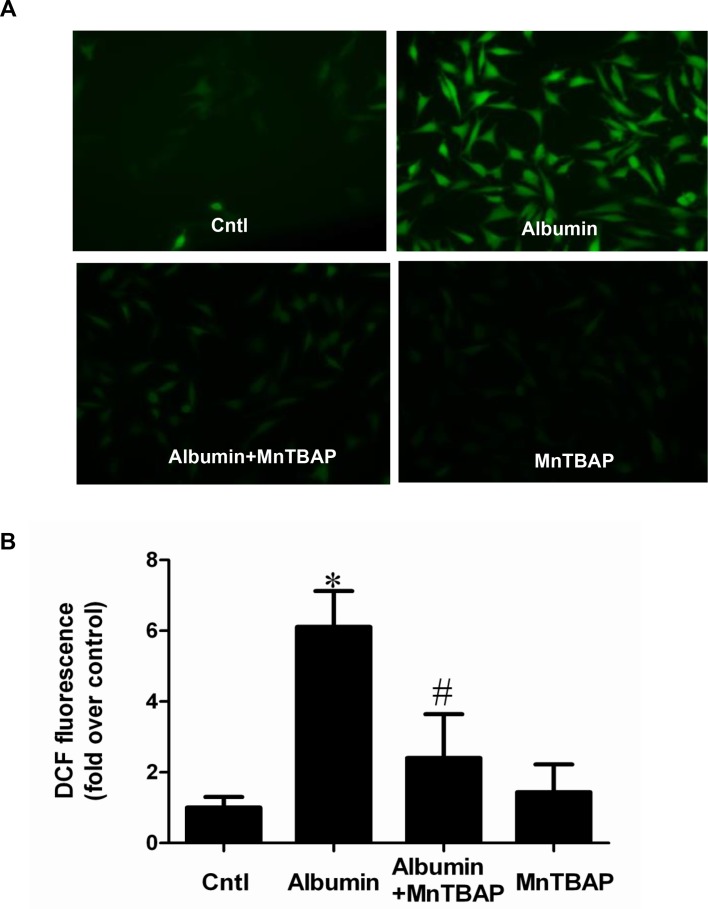
MnTBAP treatment suppressed albumin-induced ROS production in mPTCs (**A**) Representative image of DCF fluorescence. (**B**) Quantitative analysis of ROS production by DCF fluorescence. The values represent means ± SD (*n* = 6). ^*^P < 0.01 vs. control. ^#^*P <* 0.01 vs. albumin group.

**Figure 9:**
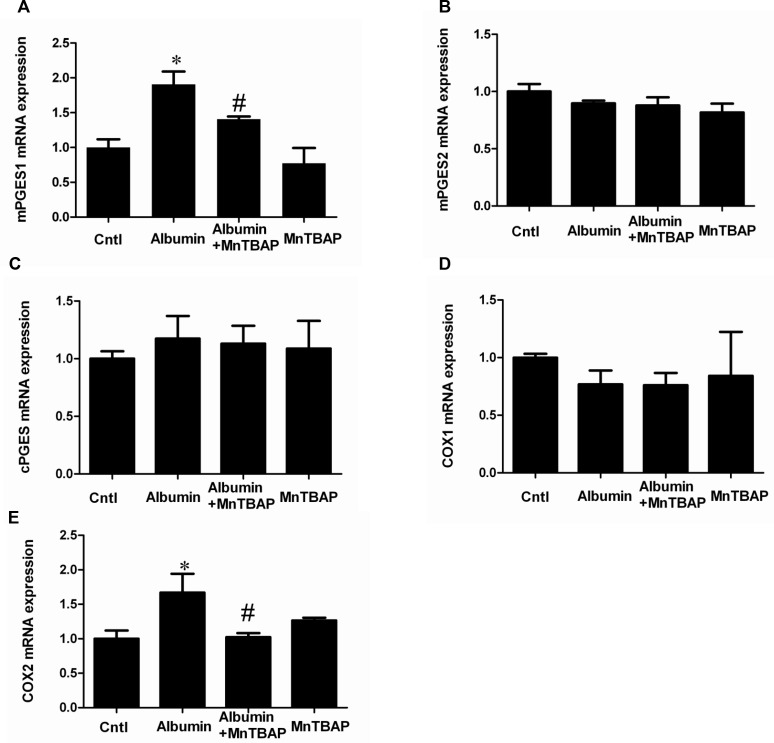
The upregulation of COX-2 and mPGES-1 mRNA expressions was revered by MnTBAP in mPTCs (**A**) qRT-PCR analysis of mPGES-1. (**B**) qRT-PCR analysis of mPGES-2. (**C**) qRT-PCR analysis of cPGES. (**D**) qRT-PCR analysis of COX-1. (**E**) qRT-PCR analysis of COX-2. The values represent means ± SD (*n* = 6). ^*^P < 0.01 vs. control. ^#^*P <* 0.01 vs. albumin group.

**Figure 10:**
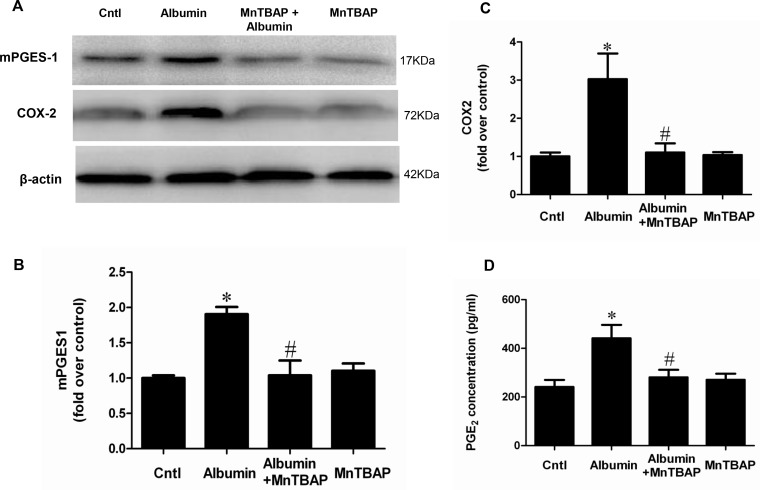
MnTBAP treatment reversed the effects of albumin on the protein expressions of COX-2 and mPGES-1 and PGE2 release in mPTCs (**A**) Western blots of mPGES-1, COX-2 and β-actin. (**B**) Densitometric analysis of mPGES-1. (**C**) Densitometric analysis of COX-2. (**D**) PGE2 release in medium. The values represent means ± SD (*n* = 6). ^*^P < 0.01 vs. control. ^#^*P <* 0.01 vs. albumin group.

Following albumin treatment, the ROS production was significantly enhanced (Figure 8A–8B). After the administration of MnTBAP, the increments of COX-2, mPGES-1, and PGE2 were significantly reversed (Figure 9A–9E and Figure 10A–10D). However, mPGES-2, cPGES, and COX-1 were not affected by albumin or MnTBAP treatment (Figure 9B–9D). These results demonstrated that the albumin effect on the activation of COX-2/mPGES-1/PGE2 cascade was through a mitochondrial oxidative stress-mediated mechanism.

## DISCUSSION

Proteinuria is a hallmark for the diagnosis of glomerular diseases. Growing evidence has also demonstrated that proteinuria is an independent causative factor mediating the progression of kidney diseases, possibly via the stimulation of the inflammatory response in tubular epithelial cells and the tubulointerstitial region. PGE2 is abundantly produced in the kidney and contributes to a number of physiological and pathological processes. Under pathological insults, PGE2 was mostly found to play a detrimental role in kidney diseases [12–14].

Treating renal epithelial cells or animals with albumin has been widely used to mimic the effect of proteinuria on kidneys. In animals, albumin overload results in the exposure of renal tubular cells to the excessive albumin filtered through the glomerular filtration barrier. Although this model cannot entirely mimic the disease status of patients with proteinuria in the clinic, it could be a suitable model for evaluating the effect of albuminuria on kidney injury because of its exclusion of non-proteinuria factors, such as diabetes, hypertension, and lipid disorders. In the present study, albumin overload for 11 days induced severe renal tubular injury accompanied by a significant increase of oxidative stress and mitochondrial morphological alteration. In line with the elevated oxidative stress, the urinary PGE2 excretion was significantly increased. After administration of a mitochondrial SOD2 mimic, the oxidative stress occurred in kidneys was robustly abolished. At the same time, the abnormality of the mitochondrial morphology in the tubular cells was strikingly improved. These results suggested that mitochondrial-derived oxidative stress strongly contributed to the albumin overload-induced mitochondrial injury and might be an important pathogenic factor in mediating proteinuria-associated kidney injury in patients.

PGE2 is an important inflammatory mediator that is involved in many kidney diseases [12, 13, 26, 27]. Blockade of the PGE2 synthetic enzymes [28] or antagonism of the PGE2 receptors substantially protects against various insults, such as diabetes, cisplatin nephropathy, adriamycin nephropathy and renal mass reduction [17–20]. Following the administration of albumin, urinary PGE2 was significantly elevated in line with the upregulation of COX-2 and mPGES-1 in kidneys. However, COX-1, mPGES-2 and cPGES were unaffected by the albumin overload. These data indicated that the activation of COX-2 and mPGES-1 might contribute to the renal PGE2 production in this model. This stimulation of COX-2/mPGES-1/PGE2 cascade was accompanied with increased oxidative stress and mitochondrial abnormality. Thus, we treated the animals with MnTBAP to evaluate the potential relationship between the activation of COX-2/mPGES-1/PGE2 cascade and mitochondrial oxidative stress. Strikingly, MnTBAP almost entirely abolished the effect of albumin on the activation of this PGE2-generating cascade, suggesting a critical role of mitochondrial oxidative stress in this process. However, due to the complicated nature of mammalian systemic responses, we could not conclude whether these effects were directly from albumin exposure or a secondary response from kidney injury. To address this, we directly exposed mouse proximal tubular cells to the albumin and reproduced the phenomenon observed *in vivo*. As shown by the data, following MnTBAP treatment, the stimulation of the COX-2/mPGES-1/PGE2 cascade was diminished in line with the attenuation of oxidative stress. These *in vitro* data clearly demonstrate a direct action of albumin on the stimulation of the COX-2/mPGES-1/PGE2 cascade via mitochondrial oxidative stress.

In summary, using both animals and *in vitro* cells, we demonstrated that albumin enhanced mitochondrial oxidative stress to stimulate COX-2/mPGES-1/PGE2 cascade in renal proximal tubular cells. These findings suggested that the inhibition of the mitochondrial oxidative stress could protect kidneys against proteinuria-associated kidney injury, and such a protective effect might be through the inactivation of COX-2/mPGES-1/PGE2 cascade to some extent.

## MATERIALS AND METHODS

### Reagents and antibodies

DMEM medium was purchased from Gibco Corporation (Carlsbad, CA, USA). Bovine serum albumin (BSA) was obtained from Sigma Chemical Co. (St. Louis, MO, USA). MnTBAP was purchased from Sigma Chemical Co. (St. Louis, MO, USA). Rabbit polyclonal antibody against COX-2 and rabbit polyclonal antibody against mPGES-1 were purchased from Cayman Chemical (USA). Rabbit antibody against β-actin was obtained from Cell Signaling Technology (Danvers, MA, USA). Peroxidase-conjugated AffiniPure goat anti-rabbit secondary antibody was from Zhongshan Gold Bridge Biotechnology (Beijing, China).

### Animal studies

For the MnTBAP experiment, 8-week-old 129/Sv male mice weighing 25–30 g were subjected to an intraperitoneal (i.p.) injection with low-endotoxin albumin (A-9430, Sigma Chemical Co., St. Louis, MO) dissolved in saline for 11 days. In brief, albumin was administered for 5 days in a stepwise incremental dose regimen, increasing from 2 mg/g body weight on the first day (D1) to the maximum dose of 10 mg/g body weight on D5, which was thereafter maintained until day 11. The control groups received a corresponding volume of saline via i.p. injection, and the albumin-treated mice received vehicle or MnTBAP (10 mg/kg/day) for 11 days.

All mice were maintained on a 12 h light-dark cycle in a temperature-controlled (19–21°C) room, were fed a standard rodent diet, and were allowed free access to drinking water. At the termination of the experiments, the mice were anesthetized with an i.p. injection of a ketamine/xylazine/atropine cocktail. Plasma and kidney samples were then immediately frozen in liquid nitrogen and stored at −80°C until use. The study protocols were reviewed and approved by the Institutional Animal Care and Use Committee at Nanjing Medical University, China.

### Cell culture studies

mPTCs, an immortalized cell line, were grown in serum-free keratinocyte medium supplemented with bovine pituitary extract and epidermal growth factor (Wisent, Canada). The cells were specifically grown at 37°C with 5% CO_2_ and subcultured at 50–80% confluence using 0.25% trypsin-0.02% EDTA (Invitrogen). In certain experiments, the cells were preincubated with MnTBAP (100 nmol/ml) for 30 min before BSA (10 mg/ml) treatment.

### Quantitative real-time PCR (qRT-PCR)

Total RNA was extracted using the TRIzol reagent (Invitrogen),. Oligonucleotides were designed using Primer3 software (available at http://frodo.wi.mit.edu/) and synthesized by Invitrogen. The sequences of the primer pairs are shown in Table 1. qRT-PCR was then used to detect the mtDNA copy number and the mRNA expression of target genes. Reverse transcription was performed using a reaction kit (Promega Reverse Transcription System) according to the manufacturer’s protocol. Real-time PCR amplification was performed using the ABI 7500 real-time PCR detection system (Foster City, CA, USA) with the SYBR Green PCR Master Mix (Applied Biosystems). The cycling conditions were 95°C for 10 min, followed by 40 cycles of 95°C for 15 s and 60°C for 1 min. The mRNA levels were normalized to GAPDH as a control and calculated using the comparative cycle threshold (ΔΔCt) method.

**Table 1:** Primer sequences for qRT-PCR

Gene symbol	Primer sequences
GAPDH	5′- GTCTTCACTACCATGGAGAAGG - 3′
	5′- TCATGGATGACCTTGGCCAG -3′
mPGES1	5′- GGATGCGCTGAAACGTGGA - 3′
	5′- CAGGAATGAGTACACGAAGCC - 3′
mPGES2	5′- CCTCGACTTCCACTCCCTG - 3′
	5′- TGAGGGCACTAATGATGACAGAG - 3′
cPGES	5′- TGTTTGCGAAAAGGAGAATCCG - 3′
	5′- CCATGTGATCCATCATCTCAGAG - 3′
COX1	5′- ATGAGTCGAAGGAGTCTCTCG - 3′
	5′- GCACGGATAGTAACAACAGGGA - 3′
COX2	5′- AACCGTGGGGAATGTATGAG - 3′
	5′- GCAGGAAGGGGATGTTGTT - 3′

### Western blotting

mPTCs were lysed using a protein lysis buffer containing 50 mM Tris, 150 mM NaCl, 10 mM EDTA, 1% Triton X-100, 200 mM sodium fluoride, and 4 mM sodium orthovanadate as a protease inhibitor (pH 7.5). Immunoblotting was then performed with primary antibodies against COX-2 (Cayman Chemical, USA, 1:500), mPGES-1 (Cayman Chemical, USA, 1:500), and β-actin (1:1000), followed by the addition of HRP-labeled secondary antibodies. The blots were visualized using the Amersham ECL detection system (Amersham, Little Chalfont, UK). Densitometric analysis was performed using Quantity One software (Bio-Rad).

### EIA assay

The concentration of PGE2 in the medium was examined using a commercial EIA kit purchased from Cayman Chemical.

### Analysis of ROS production in cells

ROS production in mPTCs was measured by DCFDA as described previously [29].

### Measurement of thiobarbituric acid-reactive substances

The measurement of plasma thiobarbituric acid-reactive substances (TBARS) was based on the formation of malondialdehyde by using a commercially available TBARS Assay kit (10009055; Cayman Chemical), according to the manufacturer’s instructions.

### Transmission electron microscopy (TEM)

Fresh kidney tissues were fixed in 1.25% glutaraldehyde/0.1 M phosphate buffer and post-fixed in 1% OsO4/0.1 M phosphate buffer. Ultrathin sections (60 nm) were then cut on a microtome, placed on copper grids, stained with uranyl acetate and lead citrate, and examined under an electron microscope (JEOL JEM-1010, Tokyo, Japan).

### Statistical analysis

All data are presented as means ± standard deviation (SD). The statistical analysis was performed using ANOVA followed by Bonferroni’s test with SPSS 13 statistical software. *P <* 0.05 was considered significant.

## Abbreviations

mPTCs: mouse proximal tubule epithelial cells
CKD: chronic kidney disease
COX-1: cyclooxygenase-1
COX-2: cyclooxygenase-2
PGE2: prostaglandin E2
mPGES-1: membrane associated PGE synthase-1
mPGES-2: membrane associated PGE synthase-2
cPGES: cytosolic PGE2 synthase
BSA: bovine serum albumin
TEM: transmission electron microscopy
TBARS: thiobarbituric acid-reactive substances
